# Universal Credit receipt among working-age patients who are accessing specialist mental health services: results from a novel data linkage study

**DOI:** 10.1136/jech-2023-221593

**Published:** 2024-06-20

**Authors:** Sharon A M Stevelink, Ioannis Bakolis, Sarah Dorrington, Johnny Downs, Ray Leal, Ira Madan, Ava Phillips, Ben Geiger, Matthew Hotopf, Nicola T Fear

**Affiliations:** 1 Department of Psychological Medicine, Institute of Psychiatry, Psychology and Neuroscience, King's College London, London, UK; 2 King's Centre for Military Health Research, Department of Psychological Medicine, Institute of Psychiatry, Psychology and Neuroscience, King's College London, London, UK; 3 South London and Maudsley NHS Foundation Trust, NIHR Maudsley Biomedical Research Centre, London, UK; 4 Department of Biostatistics and Health Informatics, Institute of Psychiatry, Psychology and Neuroscience, King's College London, London, UK; 5 Centre for Implementation Science, Health Service and Population Research Department, Institute of Psychiatry, Psychology, and Neuroscience, King's College London, London, UK; 6 Department of Occupational Health, Guy's and St Thomas' Hospitals NHS Trust, London, UK; 7 Centre for Society and Mental Health, Institute of Psychiatry, Psychology and Neuroscience, King's College London, London, UK; 8 Academic Department of Military Mental Health, Department of Psychological Medicine, Institute of Psychiatry, Psychology and Neuroscience, King's College London, London, UK

**Keywords:** EPIDEMIOLOGY, MENTAL HEALTH, PUBLIC HEALTH, HEALTH SERVICES, POLICY

## Abstract

**Background:**

In 2013, Universal Credit (UC) was introduced by the UK Government. Understanding of how UC provision is allocated among people with mental disorders, and its intersection with protected characteristics is limited. This study aimed to explore (1) how UC receipt, including UC conditionality regime, varied among users of specialist mental health services between 2013 and 2019 and (2) associations between sociodemographic and diagnostic patient characteristics and UC receipt.

**Methods:**

Working-age individuals who had accessed specialist mental health services were included if they had their mental health record data successfully linked with administrative benefits data. Associations between sociodemographic, diagnostic patient characteristics and UC receipt were explored using logistic regression models.

**Results:**

Of the 143 715 patients, 26.9% had received UC between 2013 and 2019. Four in five patients were allocated to the searching for work conditionality regime during their time on UC. Females were less likely to have received UC (adjusted OR (AOR) 0.87, 95% CI 0.85 to 0.89) than males, and UC receipt decreased with age. Black patients (AOR 1.39, 95% CI 1.34 to 1.44) and patients from mixed and multiple ethnic backgrounds (AOR 1.27, 95% CI 1.18 to 1.38) had a higher likelihood of UC receipt than White patients. UC receipt was lower among patients diagnosed with severe mental illness compared with other psychiatric diagnoses (AOR 0.74, 95% CI 0.71 to 0.77).

**Conclusion:**

One in four specialist mental health service users had received UC and a large majority were subject to conditionality. The temporality of UC conditionality and mental health service presentation needs further exploration.

**WHAT IS ALREADY KNOWN ON THIS TOPIC**

- Universal Credit (UC) was introduced in the UK in 2013 and replaced six working-age benefits (also termed legacy benefits).
- Qualitative research has indicated that the roll-out of UC has had a negative impact on the mental health of people affected. However, large-scale individual-level quantitative studies to underpin these important findings have been scarce.
- Currently, we have no knowledge about which mental disorder UC recipients may have been diagnosed with. Moreover, while data are available on certain protected characteristics of UC recipients, there are almost no data on their ethnicity. In addition, data are currently lacking on the interrelationships of these characteristics and mental disorder diagnosis in relation to UC receipt.

**WHAT THIS STUDY ADDS**

- A newly established linkage of mental health record data with administrative data on benefits receipt showed that one in four users of specialist mental health services had received UC between 2013 and 2019. Patients were often allocated to the searching for work conditionality regime.
- Female sex, older age, lower levels of deprivation and having a severe mental illness diagnosis were associated with a lower likelihood of UC receipt.
- A more varied picture was found regarding ethnicity and UC receipt.

**HOW THIS STUDY MIGHT AFFECT RESEARCH, PRACTICE OR POLICY**

- It is likely that the sociodemographic and psychiatric diagnosis profile of service users in receipt of UC will change over time once the national roll-out of UC has been finalised in the UK.
- Welfare policy must consider the substantial proportion of secondary mental health service users who are subject to UC conditionality.
- This data source provides ample future research opportunities that have the potential to impact welfare policy and service delivery directed at people with mental disorders, for example, exploring the impacts of the migration from legacy benefits to UC and the likelihood and timeliness of return to work across psychiatric diagnoses.

## Introduction

In the UK, the Department for Work and Pensions (DWP) makes payments to approximately 20 million individuals at any given time, for example, state pensions or working age, disability and ill health benefits. Of the nearly £60 billion of benefits payments made to people of working age annually, over £40 billion is accounted for by Universal Credit (UC).^1^

UC is a means-tested benefit directed at working-age people who are out of work, are unable to work or who are on a low income. As such, individuals who are employed, unemployed or economically inactive can receive UC. UC has replaced six benefits and was introduced as part of the Welfare Reform Act (2012) by the UK Government. The benefits that UC replaced are termed ‘legacy benefits’. UC was rolled out using a staggered approach from April 2013 onwards and initially only being offered to a select group of new claimants.^2^ Nationwide roll-out was expected to be finalised by the end of 2017, but this has been delayed substantially.^3^ Migration of those who are still on legacy benefits to UC is expected to be finalised by 2028/2029.^4^

A key characteristic of UC is the claimant commitment, as claimants are expected to fulfil responsibilities to continue to receive payments (referred to as ‘conditionality’). While these responsibilities are tailored to a person’s circumstances, they depend heavily on the conditionality regime that they are allocated to (which in turn depends on their personal circumstances). These regimes include (1) ‘searching for work’, (2) ‘working—with requirements’, (3) ‘no work requirements’, (4) ‘working—no requirements’, (5) ‘planning for work’ and (6) ‘preparing for work’.^5 6^ More details are in online supplemental table 1. If an individual does not fulfil the obligations outlined as part of their regime, their UC payments may be reduced or suspended. This is called a sanction and the length of the sanction depends on the reason for the sanction.^7^ Concerns have been raised that benefit recipients and particularly those affected by mental disorders are adversely impacted by the increased use of conditionality.^8^ For example, due to the fluctuating nature of certain mental disorders, it is likely that they are at an increased risk of being sanctioned as it may be more difficult to meet the conditionality requirements. Furthermore, the stress provoked by the threat of losing much needed financial support may exacerbate one’s mental disorder.

10.1136/jech-2023-221593.supp1 Supplementary data

Over the years, the number of people who are out of work due to mental ill health has increased steadily.^9^ We know that unemployed people are more likely to report poor mental health than those in employment.^10 11^ Additionally, mental health problems have been reported as a prominent health condition among those who are economically inactive due to long-term sickness.^12^ It is, therefore, likely that individuals with a mental disorder are over-represented among UC recipients particularly as people with limited capability for work or work-related ill health or a disability may be eligible to receive an extra payment, but only if they meet certain criteria assessed during a work capability assessment.^13^

Despite this, we know very little about the mental health of individuals receiving UC. We have no knowledge about which mental disorder UC claimants may have been diagnosed with. Moreover, while data are available on certain sociodemographic characteristics of UC claimants, there are almost no data on their ethnicity.^14^ This is important as people from racial and ethnic minority backgrounds face various disadvantages in the labour market and are disproportionally impacted by poor mental health.^15 16^

To address this knowledge gap, we used a novel linked data source that combined mental health record data from a large mental health service provider with administrative data concerning benefits receipt from DWP. We addressed two main aims namely (1) to describe how UC receipt, including allocation to UC conditionality regime, varies over time among users of specialist mental health services and (2) to explore the associations between sociodemographic and diagnostic patient characteristics and UC receipt.

## Methods

### Data source

A dataset was established by linking electronic mental health records from the South London and Maudsley (SLaM) National Health Service Foundation Trust with administrative records from DWP.^17^ The linked dataset included individuals who had a national insurance number and were successfully linked, including those who never applied for, or never received, benefits. Despite a high linkage rate of 92.3%, certain groups of individuals were less likely to be linked, including females, younger people and those from a non-white background. SLaM has a catchment area of 1.2 million residents covering four South London boroughs, however, it also provides national specialist mental health services.

For this study, the data coverage window for DWP administrative records ranged from 1 January 2013 to 31 December 2019. These dates were chosen as UC started to be rolled out in 2013, and the latest available full year of administrative records was 2019. Electronic health record data covered January 2007 up to December 2019. The sample was limited to working-age adults (18–66 years of age), as UC is not available to those above state pension age. Patients who died before the introduction of UC in 2013 were excluded (N=2722) (online supplemental figure 1).

### Sociodemographic and diagnostic variables

Deidentified data from SLaM electronic health records were extracted via the Clinical Records Interactive Search system.^18 19^ This included month and year of birth, ethnicity, SLaM catchment area residency, number of days a patient was active in SLaM (eg, received an episode of care), and Index of Multiple Deprivation (IMD) quintiles (2015). A patient’s first recorded primary psychiatric diagnosis was extracted based on the International Classification of Diseases (ICD)-10th revision ‘F codes’ referring to mental and behavioural disorders. In addition, we created a severe mental illness (SMI) diagnosis variable including patients who had received a primary psychiatric diagnosis that included one of the following ICD-10 ‘F codes’: F2* (schizophrenia-spectrum disorder), F30*/F31* (bipolar affective disorder) and F3* (affective disorder).^20^ Sex and vital status were extracted from administrative records.

### UC and UC conditionality regime

Benefits data were derived from DWP administrative records to inform UC receipt over the UC data coverage period (2013–2019) and on an annual basis. We also extracted the type of conditionality regime UC recipients were allocated to, namely: (1) ‘searching for work’, (2) ‘working—with requirements’, (3) ‘no work requirements’, (4) ‘working—no requirements’, (5) ‘planning for work’ and (6) ‘preparing for work’.^5^

### Statistical analysis

The data analysis protocol was preregistered (https://doi.org/10.17605/OSF.IO/EHB84). Descriptive statistics were used to describe the sociodemographic and diagnostic characteristics of the sample as well as UC receipt. We created cross-sectional snapshots of UC receipt on an annual basis (eg, 1 January 2013–31 December 2013). Additionally, we explored UC receipt over the total UC data coverage window (1 January 2013–31 December 2019). We tabulated the proportion of patients who had been in the receipt of one of the legacy benefits UC replaced. As planned, UC receipt was determined for the overall sample and restricting the sample only to patients who had lived in the SLaM catchment area. Associations between sociodemographic and diagnostic patient characteristics and UC receipt were explored using logistic regression models. Multivariable analyses were conducted simultaneously adjusting for age (continuous), sex, ethnicity, deprivation and recorded primary psychiatric diagnosis (yes/no). This analysis strategy was repeated using each of the specific UC conditionality regimes as a binary outcome of interest, restricting the sample to only those who had received UC between 2013 and 2019. Models that explored the association between SMI status and UC receipt only included patients who had received a primary psychiatric diagnosis. As per our protocol, two sensitivity analyses were conducted. The first sensitivity analysis restricted the sample to only those who had resided in the SLaM catchment area. This was done to consider the possible impact of different patient and mental health typology profiles on UC receipt among those who were referred to the specialist national mental health service provision at SLaM versus the local service provision. The second sensitivity analysis involved applying a linkage weight based on the inverse probability of being successfully linked driven by factors shown to be associated with the success of linking the mental health records with administrative records (sex, age and ethnicity).^17^ We conducted this analysis to explore the impact of patient groups that were less likely to be linked and whether this influenced our findings. The logistic regression analysis with UC receipt as the outcome was rerun using a survey command to account for the new weighting. In contrast to our data analysis protocol, it was decided not to rerun both sensitivity analyses with each of the UC conditionality regimes as an outcome of interest as the sample did not substantially differ when restricted to SLaM catchment area residents only, nor when applying the linkage weight. All statistical analyses were conducted in Stata V.17.

## Results

**Figure 1:**
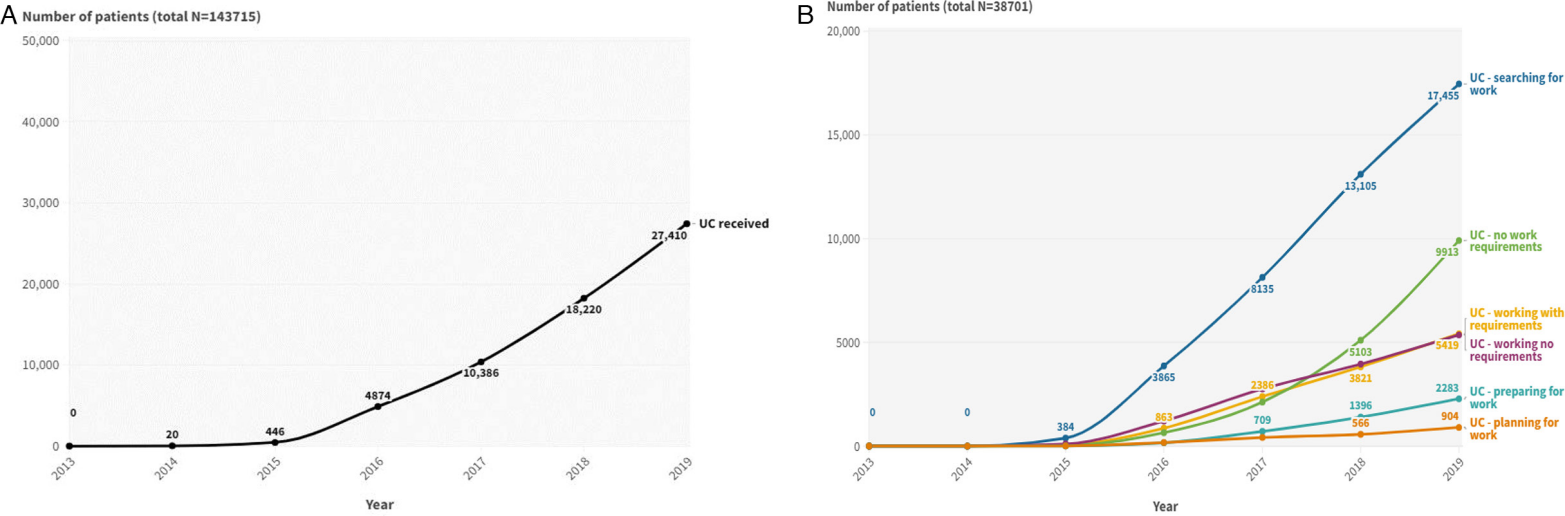
(A) Number of patients who received UC (irrespective of conditionality regime) by calendar year (N=143 715), data covering 2013–2019. (B) Number of patients who received UC by conditionality regime allocation and calendar year (N=38 701), data covering 2013–2019. UC, Universal Credit.

143 715 working-age patients were included and 26.9% had received UC between 2013 and 2019 (table 1). Of those who had received UC, most had been allocated to the ‘searching for work’ conditionality regime (80.8%) in at least one time period, followed by the ‘no work requirements’ (34.9%) and ‘working—no requirements’ regime (29.7%). UC receipt increased over time with 27 410 (19.1%) patients having received UC in 2019 (figure 1A,B). A substantial proportion of patients who had received legacy benefits had not received UC (68.3%) (table 2). UC receipt was comparable between those who did and did not reside in the SLaM catchment area (data are not shown).

**Table 1:** Profile of patients included in the study (N=143 715)

Characteristic	Total	N (%)
Sex	143 715	
Female		69 478 (48.3)
Male		74 237 (51.7)
Age (years)*	143 715	
Mean (SD)		36.7 (11.1)
18–24		24 681 (17.2)
25–34		41 376 (28.8)
35–44		36 233 (25.2)
45–54		32 332 (22.5)
55–66		9093 (6.3)
Ethnicity	119 492	
White		65 254 (54.6)
Black/African/Caribbean/Black British		20 514 (17.2)
Asian/Asian British		3123 (2.6)
Mixed/multiple racial and ethnic groups		3041 (2.5)
Other racial and ethnic minority groups		192 (0.2)
Not stated		27 368 (22.9)
Deprivation (IMD quintile)†	136 992	
First (most deprived)		43 196 (31.5)
Second		48 020 (35.1)
Third		25 565 (18.7)
Fourth		12 379 (9.0)
Fifth (least deprived)		7832 (5.7)
Resident within SLaM catchment area‡	138 669	
No		43 008 (31.0)
Yes		95 661 (69.0)
Death	143 715	
No		133 915 (93.2)
Yes		9800 (6.8)
Primary psychiatric diagnosis recorded§	143 715	
No		46 691 (32.5)
Yes		97 024 (67.5)
Total number of days active in SLaM¶	131 692	
Median days (IQR)		368 (63–1342)
Received UC	143 715	
No		105 014 (73.1)
Yes		38 701 (26.9)
UC conditionality regime** (if received UC)	38 701	
Searching for work		31 261 (80.8)
Working – with requirements		11 113 (28.7)
No work requirements		13 502 (34.9)
Working—no requirements		11 502 (29.7)
Planning for work		3812 (9.9)
Preparing for work		1823 (4.7)

*Calculated at the UC window start date (January 2013).

†IMD scores published in 2015, patient postcode used closest before or after the UC window start date (January 2013).

‡Defined as recorded at least one patient postcode within the SLaM catchment area.

§Earliest available within study window (January 2007–December 2019), based on ICD-10 ‘F codes’ only (mental and behavioural disorders) but excluding non-specific diagnoses, for example, Z*, F99*, FXX.

¶Calculated based on the first accepted referral date to SLaM within the study window and the discharge date related to the latest accepted referral to SLaM within the study window.

**Percentages will not add up to 100% as patients could have been allocated to various conditionality regimes between 2013 and 2019.

ICD-10, International Classification of Diseases, 10 Revision; IMD, Index of Multiple Deprivation; SLaM, South London and Maudsley; UC, Universal Credit.

**Table 2:** Descriptive table describing the overlap between legacy benefit receipt* (eg, housing benefit, employment and support allowance, jobseeker’s allowance and income support) and Universal Credit (UC) receipt, between 2013 and 2019 (N=143 715 of whom N=38 701 had received UC).

	Never received housing benefit, employment and support allowance, jobseeker’s allowance or income support N=38 520 N (%)	Received housing benefit, employment and support allowance, jobseeker’s allowance or income support N=105 195 N (%)
Received UC		
No	33 121 (86.0)	71 893 (68.3)
Yes	5399 (14.0)	33 302 (31.7)

*In 2013, UC was introduced to replace six benefits and tax credits namely Housing Benefit, income-related Employment and Support (ESA) Allowance, income-based Jobseeker’s Allowance (JSA), Child Tax Credit, working Tax Credit and Income Support. The linked dataset available for the current manuscript did not include information on tax credits nor the type of ESA or JSA. However, we used the available data to create a proxy for the legacy benefits that were replaced by UC as shown in the table.

After adjusting for age, sex, ethnicity, deprivation and recorded primary psychiatric diagnosis, females had a lower odds of UC receipt than males (adjusted OR (AOR) 0.87, 95% CI 0.85 to 0.89) and UC receipt decreased with older age (table 3). A trend was noted between levels of deprivation and UC receipt, whereby UC receipt decreased when patients lived in less deprived areas. Patients from a black ethnic group or a mixed ethnic group had higher odds of UC receipt compared with patients from a white or Asian ethnic group. A varied picture emerged when looking at psychiatric diagnoses (table 4). Patients diagnosed with an intellectual disability had lower odds of having received UC compared with those who had no recorded psychiatric diagnosis (AOR 0.25, 95% CI 0.21 to 0.29), whereas patients with a drug and alcohol-related disorder had higher odds of UC receipt (AOR 1.63, 95% CI 1.56 to 1.70). Patients who had an SMI diagnosis had lower odds of UC receipt compared with patients who were not diagnosed with an SMI (AOR 0.74, 95% CI 0.71 to 0.77).

**Table 3:** Overview of sociodemographic patient characteristics and UC receipt (irrespective of conditionality regime) between 2013 and 2019 (N=143 715 of whom N=38 701 had received UC).

Characteristics	Not received UC N (%)	Received UC N (%)	OR (95% CI)	P value	AOR* (95% CI)	P value
Sex						
Female	51 479 (49.0)	17 999 (46.5)	0.90 (0.88 to 0.93)	<0.001	0.87 (0.85 to 0.89)	<0.001
Male	53 535 (51.0)	20 702 (54.5)	1		1	
Age (years)†						
18–24	15 506 (14.8)	9175 (23.7)	1		1	
25–34	28 818 (27.4)	12 558 (32.5)	0.74 (0.71 to 0.76)	<0.001	0.73 (0.71 to 0.76)	<0.001
35–44	27 164 (25.9)	9069 (23.4)	0.56 (0.54 to 0.58)	<0.001	0.54 (0.52 to 0.56)	<0.001
45–54	25 744 (24.5)	6588 (17.0)	0.43 (0.42 to 0.45)	<0.001	0.39 (0.38 to 0.41)	<0.001
55–66	7782 (7.4)	1311 (3.4)	0.28 (0.27 to 0.30)	<0.001	0.27 (0.25 to 0.29)	<0.001
Ethnicity						
White	48 664 (56.2)	16 590 (50.5)	1		1	
Black/African/Caribbean/Black British	13 525 (15.6)	6989 (21.3)	1.52 (1.47 to 1.57)	<0.001	1.39 (1.34 to 1.44)	<0.001
Asian/Asian British	2320 (2.7)	803 (2.4)	1.02 (0.94 to 1.10)	0.717	0.95 (0.88 to 1.04)	0.284
Mixed/Multiple racial and ethnic groups	1998 (2.3)	1043 (3.2)	1.53 (1.42 to 1.65)	<0.001	1.27 (1.18 to 1.38)	<0.001
Other racial and ethnic minority groups	132 (0.2)	60 (0.2)	1.33 (0.98 to 1.81)	0.065	1.13 (0.82 to 1.57)	0.449
Not stated	19 968 (23.1)	7400 (22.5)	1.09 (1.05 to 1.12)	<0.001	1.00 (0.97 to 1.04)	0.821
Deprivation (IMD quintile)‡						
First (most deprived)	29 863 (29.7)	13 333 (36.5)	1		1	
Second	34 812 (34.7)	13 208 (36.2)	0.85 (0.83 to 0.87)	<0.001	0.85 (0.82 to 0.88)	<0.001
Third	19 494 (19.4)	6071 (16.6)	0.70 (0.67 to 0.72)	<0.001	0.69 (0.67 to 0.72)	<0.001
Fourth	9879 (9.8)	2500 (6.8)	0.57 (0.54 to 0.59)	<0.001	0.56 (0.53 to 0.59)	<0.001
Fifth (least deprived)	6419 (6.4)	1413 (3.9)	0.49 (0.46 to 0.52)	<0.001	0.49 (0.45 to 0.52)	<0.001

OR, AOR and their corresponding 95% CI represent an increase in odds of UC receipt.

*Adjusted for age (continuous), sex, ethnicity, deprivation and primary psychiatric diagnosis (yes/no).

†Calculated at the UC window start date (January 2013).

‡IMD scores published in 2015, patient postcode used closest before or after the UC window start date (January 2013).

AOR, adjusted OR; IMD, Index of Multiple Deprivation; UC, Universal Credit.

**Table 4:** Overview of diagnostic patient characteristics and UC receipt (irrespective of conditionality regime) between 2013 and 2019 (N=143 715 of whom N=38 701 had received UC)

Characteristics	Not received UC N (%)	Received UC N (%)	OR (95% CI)	P value	AOR* (95% CI)	P value
Primary psychiatric diagnosis Diagnosis categories† (ICD-10 codes)						
No primary psychiatric diagnosis recorded	33 741 (33.0)	12 950 (34.1)	1		1	
Schizophrenia, schizotypal and delusional disorders (F20–F29)	8341 (8.2)	2340 (6.2)	0.73 (0.70 to 0.77)	<0.001	0.63 (0.60 to 0.67)	<0.001
Severe mood disorders (ie, bipolar affective disorder, severe or moderate depressive disorders, puerperal psychosis and postnatal depression (F30–31, F32.1–32.3, F33.1–33.3, F34.0–34.1, F53.0–53.1)	3143 (3.1)	781 (2.1)	0.65 (0.60 to 0.70)	<0.001	0.68 (0.62 to 0.75)	<0.001
Anxiety, somatoform and stress-related disorders (F40–48)	14 196 (13.9)	4642 (12.2)	0.85 (0.82 to 0.89)	<0.001	0.87 (0.83 to 0.91)	<0.001
Other depressive disorders (F32.0, F32.8–32.9, F33.0, F33.4–33.9, F34.8–34.9, F38–39).	14 898 (14.6)	5534 (14.6)	0.97 (0.93 to 1.00)	0.083	1.00 (0.96 to 1.04)	0.969
Drug and alcohol-related disorders (F10–19, excluding F17)	14 694 (14.4)	7864 (20.7)	1.39 (1.35 to 1.44)	<0.001	1.63 (1.56 to 1.70)	<0.001
Personality disorders (F60–63)	2088 (2.1)	893 (2.4)	1.11 (1.03 to 1.21)	0.009	1.07 (0.97 to 1.17)	0.166
Other psychiatric disorders (including eating disorders, other perinatal psychiatric disorders and ‘unspecified mental illness’) (F50–3, F53.8–53.9, F99)	4655 (4.6)	1012 (2.7)	0.57 (0.53 to 0.61)	<0.001	0.52 (0.48 to 0.57)	<0.001
Intellectual disabilities (F70–F79)	1586 (1.6)	165 (0.4)	0.27 (0.23 to 0.32)	<0.001	0.25 (0.21 to 0.29)	<0.001
Disorders of psychological development and behavioural and emotional disorders with onset usually occurring in childhood or adolescence (F80–89, F90–98)	4858 (4.8)	1835 (4.8)	0.98 (0.93 to 1.04)	0.586	0.82 (0.77 to 0.88)	<0.001
Severe mental illness diagnosis (if yes to primary psychiatric diagnosis)						
No severe mental illness diagnosis	51 499 (75.8)	19 904 (78.7)	1		1	
Severe mental illness diagnosis (F2* (schizophrenia-spectrum disorder), F30*/F31* (bipolar affective disorder) and F3* (affective disorder)	17 393 (25.3)	5380 (21.3)	0.80 (0.77 to 0.83)	<0.001	0.74 (0.71 to 0.77)	<0.001

OR, AOR and their corresponding 95% CI represent an increase in odds of UC receipt.

*Adjusted for age (continuous), sex, ethnicity, deprivation and primary psychiatric diagnosis (yes/no).

†Earliest available within study window (January 2007–December 2019), based on ICD-10 ‘F codes’ only (mental and behavioural disorders) but excluding non-specific diagnoses, for example, Z*, F99*, FXX.

AOR, adjusted OR; ICD-10, International Classification of Diseases, 10 Revision; UC, Universal Credit.

Online supplemental table 2 provides the sociodemographic and diagnostic profile of patients by UC conditionality regime. Males were over-represented in the UC—‘searching for work’ conditionality regime whereas females were over-represented in both the UC—‘preparing for work’ and UC—‘planning for work’ regimes. Patients under the age of 35 made up at least half of the sample in each of the six regimes, as well as patients in the two most deprived IMD quintiles. A higher proportion of patients with an SMI diagnosis was found in the UC—‘no work requirements regime’ compared with the other conditionality regimes. Patient characteristics found to be associated with UC receipt for each of the six UC conditionality regimes can be found in online supplemental tables 3–8.

### Sensitivity analyses

For the first sensitivity analysis, the direction and strength of associations found based on the adjusted logistic regression between patient characteristics and UC receipt when restricting the sample to only patients who had resided in the SLaM catchment (N=95 661 of whom N=27 468 had received UC) were similar (online supplemental table 9). As planned, a second sensitivity analysis was conducted exploring the impact of a linkage weight on the associations between patient characteristics and UC receipt involving N=140 155 patients (only those who had complete data (eg, sex, age and ethnicity) to inform the linkage weight could be included)) of whom N=37 552 had received UC. The impact of the weighing on the results was negligible (online supplemental table 10).

## Discussion

Over a period of 7 years, one in four specialist mental health service users had received UC at some point, and the number of patients on UC increased steadily, as one would have expected considering the phased implementation of UC. Four in five patients had been allocated to the ‘searching for work’ conditionality regime. Furthermore, one in three patients was allocated to the ‘no work requirements’ conditionality regime meaning that they were not expected to work or search for work. National data from 2019 show a similar distribution with the highest number of people being in the ‘searching for work’ group (approximately n=900 000) followed by the ‘no work requirements’ group (approximately n=500 000). In the latest available national data (2023), this trend has been reversed with 2.1 million individuals in the ‘no work requirements’ group, followed by 1.4 million individuals in the ‘searching for work’ group.^21^ This reversal is likely due to legacy benefits claimants moving onto UC as migration of the more complex cases took place at a later stage and is ongoing.

This is also a plausible explanation as to why we found a strong negative association between a diagnosis of SMI and UC receipt, especially considering the chronic and severe nature of SMI and the impact on people’s ability to work. Indeed, when examining data regarding the receipt of legacy benefits that UC replaced, nearly 70% of our sample had received legacy benefits, and only 14% were on UC but had not received any legacy benefits (table 2). Restricting our sample to patients who never received legacy benefits, results indicated that patients with an SMI diagnosis had 1.30 (95% CI 1.20 to 1.42) odds of UC receipt compared with those with a different diagnosis. However, after adjustments this reduced to AOR 1.12 (95% CI 1.00 to 1.24) (online supplemental table 11). One in five patients who had an SMI diagnosis was in the ‘searching for work’ conditionality regime at some point, which may be surprising considering the enduring impact an SMI may have on daily life. Future explorations are needed regarding the temporality of UC receipt, conditionality regime allocations and SMI onset.

Considering that UC is a means-tested benefit we anticipated that patients living in more deprived areas were more likely to receive UC, especially considering the interrelationships between mental health and deprivation. We indeed found this. Interestingly, findings indicated a negative association between age and UC receipt, despite that in the general population UC receipt is highest among those of middle age.^21^ However, since many mental disorders have an onset in early adolescence with treatment seeking following a few years later, this might reflect our mental health service user sample.^22^ It is also possible that older patients are more likely to have remained on legacy benefits than their younger counterparts as their individual circumstances might be more complex. Hence this group would only be targeted for managed migration at a later stage during the UC roll-out.

A diverse picture was found regarding the association between ethnicity and UC receipt. Findings indicated that black patients and those from a mixed ethnic background were more likely to have received UC than white or Asian patients. This finding is probably an underestimation as we had missing data for this variable (approx. 16%), and it is expected that the proportion missingness is higher in mental health records from racial and ethnic minority patients. We also know that linkage bias is more prominent among this patient group.^23^ These limitations aside, it is well documented that certain racial and ethnic minority groups face additional inequalities in relation to the labour market, their mental health and other social determinants of health. Examples include more precarious work, such as holding lower paid and insecure jobs, a substantial mental health treatment gap, discrimination and racism.^24–26^ The DWP has indicated that ethnicity data collected as part of a UC claim has been filled in poorly, and hence not reaching their quality threshold of 70% for data release.^14^ Avenues should be explored to ensure improved completion of this important question to fully understand the possible disadvantages people from racial and ethnic minority backgrounds may face and whether they may be disproportionality impacted not only by the introduction of UC but also in the wider societal context of mental health, welfare and work.

Qualitative research has indicated that the roll-out of UC has had a negative impact on the mental health of people affected.^27–29^ However, there has been a dearth of large-scale individual-level quantitative data to underpin these important findings.^30^ The initial analyses presented here outline the mental disorders UC claimants have been diagnosed with, and the extent to which mental health service users are subject to conditionality. The novel linked data source underpinning the current study provides an important opportunity to further advance research in this field, with a particular focus on people affected by mental disorders. Nevertheless, caution is needed with regard to the generalisability of our findings, considering that SLaM covers a high-density, multiethnic urban area with substantial disparities in the distribution of income and wealth. Furthermore, the prevalence of SMI in inner London is higher compared with outer London as well as when compared with other geographical areas in England, although this is influenced by other factors including area-level deprivation.^31^

## Conclusions

A substantial number of specialist mental health service users are in receipt of UC, and this number is expected to increase once the implementation of UC has been finalised. Complex interrelationships were found between sociodemographic and diagnostic patient characteristics and UC receipt. Future research could be directed to explore the impact of work capability assessments on people with diagnosed mental disorders, the likelihood of return to work across psychiatric diagnoses, as well as the mental health and occupational impact of the migration from legacy benefits to UC, and whether groups of patients, for example, those from racial and ethnic minority backgrounds are disproportionally impacted.

## Data Availability

No data are available. Data are not publicly available. Access to deidentified data can be applied for via the NIHRMaudsley Biomedical Research Centre at the South London and Maudsley NHS Foundation Trust, on reasonable request. Requests for data will be considered on a case-by-case basis, given the sensitive nature of the data and access will only be granted if approval is given by the Work and Health Screening Panel and other governance requirements are fulfilled. For more information, please contact: cris.administrator@slam.nhs.uk.
