# Supplementary material for: Universal Credit receipt among working-age patients who are accessing specialist mental health services: results from a novel data linkage study
Source: J Epidemiol Community Health. 2024 Jun 20;78(9):592–8. doi: 10.1136/jech-2023-221593 (PMC11347967; doi:10.1136/jech-2023-221593)
Supplement: Supplementary data [file jech-2023-221593supp001.pdf]

BMJ Publishing Group Limited (BMJ) disclaims all liability and responsibility arising from any reliance
placed on this supplemental material which has been supplied by the author(s)

Supplementary Figure 1: Flow diagram outlining patient in and exclusion criteria resulting in the final sample size of N=143715 working age adults.

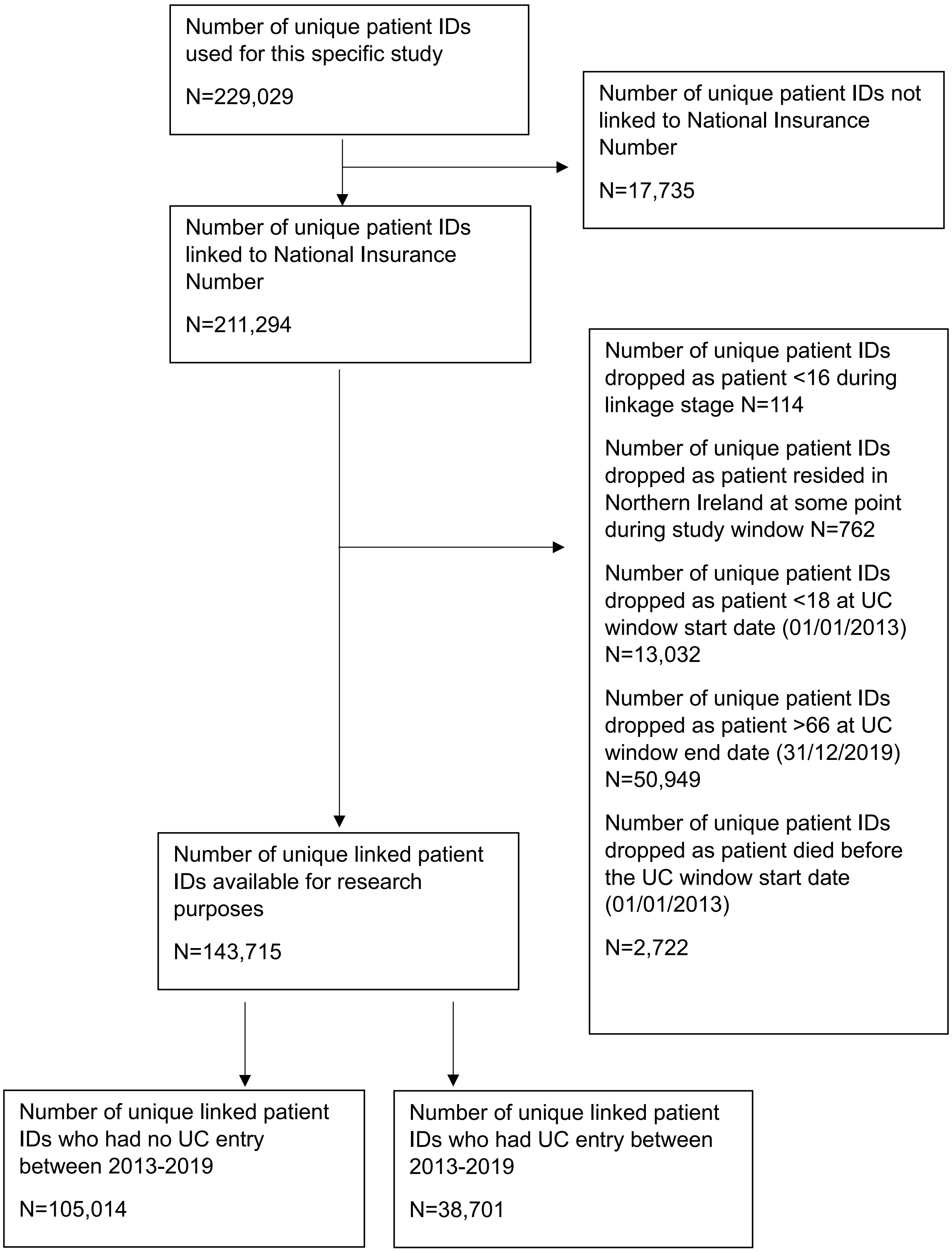

Supplementary Table 1: Overview of the UC conditionality regimes and work-related activities.

**Conditionality Regime**	**Description**
Searching for work	Not working, or with very low earnings. Claimant is required to take action to secure work - or more / better paid work. The Work Coach supports them to plan their work search and preparation activity.
Working - with requirements	In work but could earn more, or not working but has a partner with low earnings
No work requirements	Not expected to work at present. Health or caring responsibility prevents claimant from working or preparing for work.
Working – no requirements	Individual or household earnings over the level at which conditionality applies. Required to inform DWP of changes of circumstances, particularly if at risk of decreasing earnings or losing job.
Planning for work	Expected to work in the future. Lone parent / lead carer of child aged 1[(a)]. Claimant required to attend periodic interviews to plan for their return to work.
Preparing for work	Expected to start preparing for future even with limited capability for work at the present time or a child aged 2[(b)], the claimant is expected to take reasonable steps to prepare for work including Work Focused Interview.
a) Aged 1-2, prior to April 2017 b) Aged 3-4, prior to April 2017	

Reprinted from the Department for Work and Pensions Stat-Xplore webpage detailing the Dataset: People on Universal Credit (DWP).

BMJ Publishing Group Limited (BMJ) disclaims all liability and responsibility arising from any reliance placed on this supplemental material which has been supplied by the author(s)

Supplementary Table 2: Sociodemographic and diagnostic characteristics of patients by UC conditionality regime, data covering 2013-2019 (N=38701 had received UC).

**Characteristics**	**Allocated to UC - searching for work (N=31261) N (%)**	**Allocated to UC - working with requirements (N=11113) N (%)**	**Allocated to UC - no work requirements (N=13052) N (%)**	**Allocated to UC - working no requirements (N=11502) N (%)**	**Allocated to UC - preparing for work (N=3812) N (%)**	**Allocated to UC - planning for work (N=1823) N (%)**
**Sex**						
Female	13294 (42.5)	5621 (50.6)	7317 (54.2)	5993 (52.1)	2224 (58.3)	1565 (85.9)
Male	17967 (57.5)	5492 (49.4)	6185 (45.8)	5509 (47.9)	1588 (41.7)	258 (14.2)
**Age (years)**^						
18-24	7353 (23.5)	2864 (25.8)	3067 (22.7)	3115 (27.1)	948 (24.9)	797 (43.7)
25-34	9850 (31.5)	3711 (33.4)	4265 (31.6)	4207 (36.6)	1252 (32.8)	843 (46.2)
35-44	7433 (23.8)	2625 (23.6)	2973 (22.0)	2571 (22.4)	854 (22.4)	159 (8.7)
45-54	5519 (17.7)	1677 (15.1)	2562 (19.0)	1426 (12.4)	623 (16.3)	<30 (<1.5)
55-66	1106 (3.5)	236 (2.1)	635 (4.7)	183 (1.6)	135 (3.5)	<5 (<1.0)
**Ethnicity**						
White	13382 (50.2)	4292 (46.0)	6046 (52.0)	4590 (47.2)	1659 (51.0)	632 (41.9)
Black/African/Caribbean/ Black British	5807 (21.8)	2193 (23.5)	2510 (21.6)	2170 (22.3)	667 (20.5)	419 (27.8)
Asian/Asian British	642 (2.4)	254 (2.7)	307 (2.6)	235 (2.4)	85 (2.6)	<50 (<4.0)
Mixed/Multiple racial and ethnic groups	872 (3.3)	292 (3.1)	365 (3.1)	281 (2.9)	117 (3.6)	56 (3.7)
Other racial and ethnic minority groups	50 (0.2)	16 (0.2)	27 (0.2)	14 (0.1)	8 (0.3)	<5 (<1.0)
Not stated	5884 (22.1)	2277 (24.4)	2374 (20.4)	2436 (25.1)	716 (22.0)	352 (23.3)
**Deprivation (IMD quintile)**¥						
First (most deprived)	10793 (36.8)	3958 (37.2)	4994 (39.1)	3818 (34.5)	1418 (38.9)	692 (39.1)

Second	10653 (36.3)	3841 (36.1)	4600 (36.0)	4037 (36.5)	1354 (37.1)	673 (38.0)
Third	4859 (16.6)	1716 (16.1)	1927 (15.1)	1930 (17.5)	584 (16.0)	264 (14.9)
Fourth	1952 (6.7)	708 (6.7)	822 (6.4)	775 (7.0)	195 (5.4)	98 (5.5)
Fifth (least deprived)	1103 (3.8)	408 (3.8)	440 (3.4)	495 (4.5)	96 (2.6)	45 (2.5)
**Primary psychiatric diagnosis Diagnosis categories**$ *(ICD-10 codes)*						
No primary psychiatric diagnosis recorded	10344 (33.7)	4066 (37.2)	3974 (30.1)	4258 (37.6)	1221 (32.5)	674 (37.3)
Schizophrenia, schizotypal and delusional disorders (F20-F29)	1986 (6.5)	626 (5.7)	1121 (8.5)	522 (4.6)	94 (2.5)	48 (2.7)
Severe mood disorders (i.e. bipolar affective disorder, severe or moderate depressive disorders, puerperal psychosis and postnatal depression (F30-31, F32.1-32.3, F33.1-33.3, F34.0-34.1, F53.0-53.1)	621 (2.0)	226 (2.1)	331 (2.5)	246 (2.2)	64 (1.7)	25 (1.4)
Anxiety, somatoform and stress-related disorders (F40-48)	3507 (11.4)	1477 (13.5)	1739 (13.2)	1581 (14.0)	566 (15.1)	288 (15.9)
Other depressive disorders (F32.0, F32.8-32.9, F33.0, F33.4-33.9, F34.8-34.9, F38-39).	4253 (13.9)	1705 (15.6)	2261 (17.1)	1823 (16.1)	722 (19.2)	411 (22.7)
Drug and alcohol-related disorders (F10-19, excluding F17)	6980 (22.7)	1696 (15.5)	2371 (18.0)	1624 (14.3)	743 (19.8)	158 (8.7)
Personality disorders (F60-63)	709 (2.3)	252 (2.3)	424 (3.2)	235 (2.1)	83 (2.2)	<40 (<3.0)
Other psychiatric disorders (including eating disorders, other perinatal psychiatric disorders, and	722 (2.4)	290 (2.7)	294 (2.2)	434 (3.8)	107 (2.9)	78 (4.3)

'unspecified mental illness') (F50-3, F53.8-53.9, F99)						
Intellectual disabilities (F70-F79)	133 (0.4)	27 (0.3)	89 (0.7)	12 (0.1)	16 (0.4)	<5 (<1.0)
Disorders of psychological development and behavioural and emotional disorders with onset usually occurring in childhood or adolescence (F80-89, F90-98)	1459 (4.8)	563 (5.2)	601 (4.6)	587 (5.2)	144 (3.8)	84 (4.6)
**Severe mental illness diagnosis** *(if yes to primary psychiatric diagnosis)*						
No severe mental illness diagnosis	16206 (78.9)	5387 (78.0)	6900 (74.0)	5655 (79.4)	2118 (82.5)	912 (79.3)
Severe mental illness diagnosis (F2* (schizophrenia-spectrum disorder), F30*/F31* (bipolar affective disorder) and F3* (affective disorder)	4339 (21.1)	1521 (22.0)	2426 (26.0)	1471 (20.6)	448 (17.5)	238 (20.7)

IMD: Index of Multiple Deprivation. UC: Universal Credit. ^ Calculated at the UC window start date (January 2013). ¥ IMD scores published in 2015, patient postcode used closest before or after the UC window start date (January 2013). $ Earliest available within study window (January 2007 to December 2019), based on ICD-10 'F codes' only (mental and behavioural disorders) but excluding non-specific diagnoses, for example, Z*, F99*, FXX. Numbers may not add up due to missing data and suppression of cell counts due to small numbers.

Supplementary Table 3: Overview of patients by UC conditionality regime - searching for work (N=38701 of whom N=31261 were allocated to the UC - searching for work conditionality regime). Odds ratio (OR), Adjusted odds ratios (AOR) and their corresponding 95% intervals (CI) represent increase in odds of UC receipt.

**Characteristics**	**Never allocated UC - searching for work 2013-2019 N (%)**	**Allocated UC - searching for work 2013-2019 N (%)**	**OR (95% CI)**	**p-value**	**AOR# (95% CI)**	**p-value**
**Sex**						
Female	4705 (63.2)	13294 (42.5)	0.43 (0.41-0.45)	<0.001	0.42 (0.40-0.45)	<0.001
Male	2735 (36.8)	17967 (57.5)	1		1	
**Age (years)^**						
18-24	1822 (24.5)	7353 (23.5)	1		1	
25-34	2708 (36.4)	9850 (31.5)	0.90 (0.84-0.96)	0.002	0.87 (0.80-0.93)	<0.001
35-44	1636 (22.0)	7433 (23.8)	1.13 (1.05-1.21)	0.002	1.05 (0.97-1.14)	0.238
45-54	1069 (14.4)	5519 (17.7)	1.28 (1.18-1.39)	<0.001	1.14 (1.04-1.26)	0.005
55-66	205 (2.8)	1106 (3.5)	1.34 (1.14-1.57)	<0.001	1.24 (1.04-1.48)	0.017
**Ethnicity**						
White	3208 (51.3)	13382 (50.2)	1		1	
Black/African/Caribbean/ Black British	1182 (18.9)	5807 (21.8)	1.18 (1.09-1.27)	<0.001	1.24 (1.15-1.34)	<0.001
Asian/Asian British	161 (2.6)	642 (2.4)	0.96 (0.80-1.14)	0.618	1.02 (0.85-1.22)	0.858
Mixed/Multiple racial and ethnic groups	171 (2.7)	872 (3.3)	1.22 (1.03-1.45)	0.019	1.38 (1.16-1.65)	<0.001
Other racial and ethnic minority groups	10 (0.2)	50 (0.2)	1.20 (0.61-2.37)	0.602	1.41 (0.66-3.01)	0.376
Not stated	1516 (24.3)	5884 (22.1)	0.93 (0.87-1.00)	0.039	0.97 (0.90-1.04)	0.415
**Deprivation (IMD quintile)¥**						
First (most deprived)	2540 (35.5)	10793 (36.8)	1		1	
Second	2555 (35.7)	10653 (36.3)	0.98 (0.92-1.04)	0.545	0.97 (0.91-1.04)	0.459

Third	1212 (16.9)	4859 (16.6)	0.94 (0.87-1.02)	0.135	0.95 (0.87-1.03)	0.221
Fourth	548 (7.7)	1952 (6.7)	0.84 (0.76-0.93)	0.001	0.84 (0.75-0.94)	0.003
Fifth (least deprived)	310 (4.3)	1103 (3.8)	0.84 (0.73-0.96)	0.009	0.84 (0.73-0.97)	0.018
**Primary psychiatric diagnosis** **Diagnosis categories**$ *(ICD-10 codes)*						
No primary psychiatric diagnosis recorded	2606 (35.7)	10344 (33.7)	1		1	
Schizophrenia, schizotypal and delusional disorders (F20-F29)	354 (4.9)	1986 (6.5)	1.41 (1.25-1.60)	<0.001	1.19 (1.03-1.37)	0.019
Severe mood disorders (i.e. bipolar affective disorder, severe or moderate depressive disorders, puerperal psychosis and postnatal depression (F30-31, F32.1-32.3, F33.1-33.3, F34.0-34.1, F53.0-53.1)	160 (2.2)	621 (2.0)	0.98 (0.82-1.17)	0.81	1.00 (0.81-1.23)	0.979
Anxiety, somatoform and stress-related disorders (F40-48)	1135 (15.5)	3507 (11.4)	0.78 (0.72-0.84)	<0.001	0.82 (0.75-0.90)	<0.001
Other depressive disorders (F32.0, F32.8-32.9, F33.0, F33.4-33.9, F34.8-34.9, F38-39).	1281 (17.5)	4253 (13.9)	0.84 (0.78-0.90)	<0.001	0.93 (0.85-1.02)	0.115
Drug and alcohol-related disorders (F10-19, excluding F17)	884 (12.1)	6980 (22.7)	1.99 (1.83-2.16)	<0.001	1.74 (1.58-1.92)	<0.001
Personality disorders (F60-63)	184 (2.5)	709 (2.3)	0.97 (0.82-1.15)	0.729	1.02 (0.84-1.23)	0.845
Other psychiatric disorders (including eating disorders, other perinatal psychiatric disorders, and 'unspecified mental illness') (F50-3, F53.8-53.9, F99)	290 (4.0)	722 (2.4)	0.63 (0.54-0.72)	<0.001	0.85 (0.72-1.00)	0.053
Intellectual disabilities (F70-F79)	32 (0.4)	133 (0.4)	1.05 (0.71-1.54)	0.816	0.95 (0.63-1.42)	0.793
Disorders of psychological development and behavioural and emotional disorders with onset usually occurring in childhood or adolescence (F80-89, F90-98)	376 (5.2)	1459 (4.8)	0.98 (0.87-1.10)	0.714	0.92 (0.81-1.05)	0.196

**Severe mental illness diagnosis** *(if yes to primary psychiatric diagnosis)*						
No severe mental illness diagnosis	3698 (78.0)	16206 (78.9)	1		1	
Severe mental illness diagnosis (F2* (schizophrenia-spectrum disorder), F30*/F31* (bipolar affective disorder) and F3* (affective disorder)	1041 (22.0)	4339 (21.1)	0.95 (0.88-1.03)	0.199	0.93 (0.85-1.02)	0.137

AOR: Adjusted Odds Ratio. IMD: Index of Multiple Deprivation. OR: Odds ratio. UC: Universal Credit. ^ Calculated at the UC window start date (January 2013). ¥ IMD scores published in 2015, patient postcode used closest before or after the UC window start date (January 2013). $ Earliest available within study window (January 2007 to December 2019), based on ICD-10 ‘F codes’ only (mental and behavioural disorders) but excluding non-specific diagnoses, for example, Z*, F99*, FXX. # Adjusted for age (continuous), sex, ethnicity, deprivation and primary psychiatric diagnosis (yes/no).

Supplementary Table 4: Overview of patients by UC conditionality regime – working with requirements (N=38701 of whom N=11113 were allocated to the UC - working with requirements conditionality regime). Odds ratio (OR), Adjusted odds ratios (AOR) and their corresponding 95% intervals (CI) represent increase in odds of UC receipt.

**Characteristics**	**Never allocated UC – working with requirements 2013-2019 N (%)**	**Allocated UC - working with Requirements 2013-2019 N (%)**	**OR (95% CI)**	**p-value**	**AOR# (95% CI)**	**p-value**
**Sex**						
Female	12378 (44.9)	5621 (50.6)	1.26 (1.20-1.31)	<0.001	1.23 (1.18-1.30)	<0.001
Male	15210 (55.1)	5492 (49.4)	1		1	
**Age (years)**^						
18-24	6311 (22.9)	2864 (25.8)	1		1	
25-34	8847 (32.1)	3711 (33.4)	0.92 (0.87-0.98)	0.008	0.92 (0.87-0.99)	0.016
35-44	6444 (23.4)	2625 (23.6)	0.90 (0.84-0.96)	0.001	0.92 (0.86-0.99)	0.022
45-54	4911 (17.8)	1677 (15.1)	0.75 (0.70-0.81)	<0.001	0.75 (0.69-0.81)	<0.001
55-66	1075 (3.9)	236 (2.1)	0.48 (0.42-0.56)	<0.001	0.50 (0.42-0.59)	<0.001
**Ethnicity**						
White	12298 (52.2)	4292 (46.0)	1		1	
Black/African/Caribbean/ Black British	4796 (20.4)	2193 (23.5)	1.31 (1.23-1.39)	<0.001	1.28 (1.20-1.36)	<0.001
Asian/Asian British	549 (2.3)	254 (2.7)	1.33 (1.14-1.54)	<0.001	1.29 (1.10-1.51)	0.002
Mixed/Multiple racial and ethnic groups	751 (3.2)	292 (3.1)	1.11 (0.97-1.28)	0.129	1.05 (0.91-1.22)	0.465
Other racial and ethnic minority groups	44 (0.2)	16 (0.2)	1.04 (0.59-1.85)	0.888	0.98 (0.53-1.80)	0.945
Not stated	5123 (21.7)	2277 (24.4)	1.27 (1.20-1.35)	<0.001	1.17 (1.09-1.24)	<0.001
**Deprivation (IMD quintile)**¥						
First (most deprived)	9375 (36.2)	3958 (37.2)	1		1	

Second	9367 (36.2)	3841 (36.1)	0.97 (0.92-1.02)	0.279	0.98 (0.93-1.04)	0.510
Third	4355 (16.8)	1716 (16.1)	0.93 (0.87-1.00)	0.044	0.94 (0.87-1.01)	0.101
Fourth	1792 (6.9)	708 (6.7)	0.94 (0.85-1.03)	0.169	0.98 (0.89-1.09)	0.733
Fifth (least deprived)	1005 (3.9)	408 (3.8)	0.96 (0.85-1.09)	0.525	0.96 (0.85-1.10)	0.567
**Primary psychiatric diagnosis Diagnosis categories**$ *(ICD-10 codes)*						
No primary psychiatric diagnosis recorded	8884 (32.8)	4066 (37.2)	1		1	
Schizophrenia, schizotypal and delusional disorders (F20-F29)	1714 (6.3)	626 (5.7)	0.80 (0.72-0.88)	<0.001	0.75 (0.67-0.84)	<0.001
Severe mood disorders (i.e. bipolar affective disorder, severe or moderate depressive disorders, puerperal psychosis and postnatal depression (F30-31, F32.1-32.3, F33.1-33.3, F34.0-34.1, F53.0-53.1)	555 (2.1)	226 (2.1)	0.89 (0.76-1.04)	0.150	0.87 (0.72-1.04)	0.127
Anxiety, somatoform and stress-related disorders (F40-48)	3165 (11.7)	1477 (13.5)	1.02 (0.95-1.10)	0.597	0.96 (0.89-1.05)	0.362
Other depressive disorders (F32.0, F32.8-32.9, F33.0, F33.4-33.9, F34.8-34.9, F38-39).	3829 (14.1)	1705 (15.6)	0.97 (0.91-1.04)	0.429	0.95 (0.88-1.03)	0.182
Drug and alcohol-related disorders (F10-19, excluding F17)	6168 (22.8)	1696 (15.5)	0.60 (0.56-0.64)	<0.001	0.63 (0.59-0.69)	<0.001
Personality disorders (F60-63)	641 (2.4)	252 (2.3)	0.86 (0.74-1.00)	0.048	0.85 (0.72-1.01)	0.065
Other psychiatric disorders (including eating disorders, other perinatal psychiatric disorders, and ‘unspecified mental illness’) (F50-3, F53.8-53.9, F99)	722 (2.7)	290 (2.7)	0.88 (0.76-1.01)	0.070	0.81 (0.69-0.95)	0.011
Intellectual disabilities (F70-F79)	138 (0.5)	27 (0.3)	0.43 (0.28-0.65)	<0.001	0.45 (0.29-0.68)	<0.001

Disorders of psychological development and behavioural and emotional disorders with onset usually occurring in childhood or adolescence (F80-89, F90-98)	1272 (4.7)	563 (5.2)	0.97 (0.87-1.08)	0.536	0.94 (0.84-1.05)	0.300
**Severe mental illness diagnosis** *(if yes to primary psychiatric diagnosis)*						
No severe mental illness diagnosis	14517 (79.0)	5387 (78.0)	1		1	
Severe mental illness diagnosis (F2* (schizophrenia-spectrum disorder), F30*/F31* (bipolar affective disorder) and F3* (affective disorder)	3859 (21.0)	1521 (22.0)	1.06 (0.99-1.14)	0.078	1.00 (0.92-1.08)	0.921

AOR: Adjusted Odds Ratio. IMD: Index of Multiple Deprivation. OR: Odds ratio. UC: Universal Credit. ^ Calculated at the UC window start date (January 2013). ¥ IMD scores published in 2015, patient postcode used closest before or after the UC window start date (January 2013). $ Earliest available within study window (January 2007 to December 2019), based on ICD-10 'F codes' only (mental and behavioural disorders) but excluding non-specific diagnoses, for example, Z*, F99*, FXX. # Adjusted for age (continuous), sex, ethnicity, deprivation and primary psychiatric diagnosis (yes/no).

Supplementary Table 5: Overview of patients by UC conditionality regime – no work requirements (N=38701 of whom N=13052 were allocated to the UC - no work requirements conditionality regime). Odds ratio (OR), Adjusted odds ratios (AOR) and their corresponding 95% intervals (CI) represent increase in odds of UC receipt.

**Characteristics**	**Never allocated UC - no work requirements 2013-2019 N (%)**	**Allocated UC - no work requirements 2013-2019 N (%)**	**OR (95% CI)**	**p-value**	**AOR# (95% CI)**	**p-value**
**Sex**						
Female	10682 (42.4)	7317 (54.2)	1.61 (1.54-1.68)	<0.001	1.65 (1.57-1.73)	<0.001
Male	14517 (57.6)	6185 (45.8)	1		1	
**Age (years)**^						
18-24	6108 (24.2)	3067 (22.7)	1		1	
25-34	8293 (32.9)	4265 (31.6)	1.02 (0.97-1.08)	0.413	1.04 (0.97-1.11)	0.247
35-44	6096 (24.2)	2973 (22.0)	0.97 (0.91-1.03)	0.354	0.97 (0.91-1.05)	0.475
45-54	4026 (16.0)	2562 (19.0)	1.27 (1.19-1.35)	<0.001	1.28 (1.19-1.38)	<0.001
55-66	676 (2.7)	635 (4.7)	1.87 (1.66-2.10)	<0.001	1.97 (1.73-2.24)	<0.001
**Ethnicity**						
White	10544 (49.6)	6046 (52.0)	1		1	
Black/African/Caribbean/ Black British	4479 (21.1)	2510 (21.6)	0.98 (0.92-1.04)	0.440	0.92 (0.87-0.98)	0.010
Asian/Asian British	496 (2.3)	307 (2.6)	1.08 (0.93-1.25)	0.304	1.00 (0.86-1.16)	0.961
Mixed/Multiple racial and ethnic groups	678 (3.2)	365 (3.1)	0.94 (0.82-1.07)	0.346	0.88 (0.76-1.00)	0.059
Other racial and ethnic minority groups	33 (0.2)	27 (0.2)	1.43 (0.86-2.38)	0.172	1.44 (0.84-2.46)	0.185
Not stated	5026 (23.6)	2374 (20.4)	0.82 (0.78-0.87)	<0.001	0.90 (0.85-0.96)	0.001
**Deprivation (IMD quintile)**¥						
First (most deprived)	8339 (35.1)	4994 (39.1)	1		1	
Second	8608 (36.3)	4600 (36.0)	0.89 (0.85-0.94)	<0.001	0.90 (0.85-0.95)	<0.001

Third	4144 (17.5)	1927 (15.1)	0.78 (0.73-0.83)	<0.001	0.77 (0.72-0.83)	<0.001
Fourth	1678 (7.1)	822 (6.4)	0.82 (0.75-0.90)	<0.001	0.82 (0.74-0.90)	<0.001
Fifth (least deprived)	973 (4.1)	440 (3.4)	0.76 (0.67-0.85)	<0.001	0.77 (0.67-0.87)	<0.001
**Primary psychiatric diagnosis** **Diagnosis categories**$ *(ICD-10 codes)*						
No primary psychiatric diagnosis recorded	8976 (36.2)	3974 (30.1)	1		1	
Schizophrenia, schizotypal and delusional disorders (F20-F29)	1219 (4.9)	1121 (8.5)	2.08 (1.90-2.27)	<0.001	2.25 (2.02-2.50)	<0.001
Severe mood disorders (i.e. bipolar affective disorder, severe or moderate depressive disorders, puerperal psychosis and postnatal depression (F30-31, F32.1-32.3, F33.1-33.3, F34.0-34.1, F53.0-53.1)	450 (1.8)	331 (2.5)	1.66 (1.43-1.92)	<0.001	1.50 (1.26-1.78)	<0.001
Anxiety, somatoform and stress-related disorders (F40-48)	2903 (11.7)	1739 (13.2)	1.35 (1.26-1.45)	<0.001	1.25 (1.15-1.36)	<0.001
Other depressive disorders (F32.0, F32.8-32.9, F33.0, F33.4-33.9, F34.8-34.9, F38-39).	3273 (13.2)	2261 (17.1)	1.56 (1.46-1.67)	<0.001	1.38 (1.28-1.49)	<0.001
Drug and alcohol-related disorders (F10-19, excluding F17)	5493 (22.1)	2371 (18.0)	0.97 (0.92-1.04)	0.414	1.04 (0.96-1.11)	0.352
Personality disorders (F60-63)	469 (1.9)	424 (3.2)	2.04 (1.78-2.34)	<0.001	1.93 (1.66-2.26)	<0.001
Other psychiatric disorders (including eating disorders, other perinatal psychiatric disorders, and ‘unspecified mental illness’) (F50-3, F53.8-53.9, F99)	718 (2.9)	294 (2.2)	0.92 (0.80-1.06)	0.277	0.82 (0.70-0.96)	0.014
Intellectual disabilities (F70-F79)	76 (0.3)	89 (0.7)	2.65 (1.94-3.60)	<0.001	2.40 (1.73-3.33)	<0.001
Disorders of psychological development and behavioural and emotional disorders with onset usually occurring in childhood or adolescence (F80-89, F90-98)	1234 (5.0)	601 (4.6)	1.10 (0.99-1.22)	0.073	1.27 (1.14-1.42)	<0.001

**Severe mental illness diagnosis** *(if yes to primary psychiatric diagnosis)*						
No severe mental illness diagnosis	13004 (81.5)	6900 (74.0)	1		1	
Severe mental illness diagnosis (F2* (schizophrenia-spectrum disorder), F30*/F31* (bipolar affective disorder) and F3* (affective disorder)	2954 (18.5)	2426 (26.0)	1.55 (1.46-1.65)	<0.001	1.54 (1.43-1.65)	<0.001

AOR: Adjusted Odds Ratio. IMD: Index of Multiple Deprivation. OR: Odds ratio. UC: Universal Credit. ^ Calculated at the UC window start date (January 2013). ¥ IMD scores published in 2015, patient postcode used closest before or after the UC window start date (January 2013). $ Earliest available within study window (January 2007 to December 2019), based on ICD-10 'F codes' only (mental and behavioural disorders) but excluding non-specific diagnoses, for example, Z*, F99*, FXX. # Adjusted for age (continuous), sex, ethnicity, deprivation and primary psychiatric diagnosis (yes/no).

Supplementary Table 6: Overview of patients by UC conditionality regime – working no requirements (N=38701 of whom N=11502 were allocated to the UC - working no requirements conditionality regime). Odds ratio (OR), Adjusted odds ratios (AOR) and their corresponding 95% intervals (CI) represent increase in odds of UC receipt.

Characteristics	Never allocated UC – working no requirements 2013-2019 N (%)	Allocated UC – working no requirements 2013-2019 N (%)	OR (95% CI)	p-value	AOR# (95% CI)	p-value
**Sex**						
Female	12006 (44.1)	5993 (52.1)	1.38 (1.32-1.44)	<0.001	1.31 (1.24-1.37)	<0.001
Male	15193 (55.9)	5509 (47.9)	1		1	
**Age (years)^**						
18-24	6060 (22.3)	3115 (27.1)	1		1	
25-34	8351 (30.7)	4207 (36.6)	0.98 (0.93-1.04)	0.488	0.99 (0.93-1.06)	0.777
35-44	6498 (23.9)	2571 (22.4)	0.77 (0.72-0.82)	<0.001	0.80 (0.75-0.86)	<0.001
45-54	5162 (18.9)	1426 (12.4)	0.54 (0.50-0.58)	<0.001	0.56 (0.51-0.60)	<0.001
55-66	1128 (4.2)	183 (1.6)	0.32 (0.27-0.37)	<0.001	0.33 (0.28-0.40)	<0.001
**Ethnicity**						
White	12000 (51.8)	4590 (47.2)	1		1	
Black/African/Caribbean/ Black British	4819 (20.8)	2170 (22.3)	1.18 (1.11-1.25)	<0.001	1.16 (1.09-1.24)	<0.001
Asian/Asian British	568 (2.5)	235 (2.4)	1.08 (0.93-1.26)	0.323	1.04 (0.89-1.23)	0.601
Mixed/Multiple racial and ethnic groups	762 (3.3)	281 (2.9)	0.96 (0.84-1.11)	0.611	0.86 (0.75-1.00)	0.049
Other racial and ethnic minority groups	46 (0.2)	14 (0.1)	0.80 (0.44-1.45)	0.455	0.80 (0.43-1.51)	0.496
Not stated	4964 (21.4)	2436 (25.1)	1.28 (1.21-1.36)	<0.001	1.15 (1.08-1.22)	<0.001
**Deprivation (IMD quintile)¥**						
First (most deprived)	9515 (37.4)	3818 (34.5)	1		1	
Second	9171 (36.0)	4037 (36.5)	1.10 (1.04-1.16)	0.001	1.10 (1.04-1.17)	0.001

Third	4141 (16.3)	1930 (17.5)	1.16 (1.09-1.24)	<0.001	1.18 (1.10-1.27)	<0.001
Fourth	1725 (6.8)	775 (7.0)	1.12 (1.02-1.23)	0.017	1.11 (1.00-1.23)	0.040
Fifth (least deprived)	918 (3.6)	495 (4.5)	1.34 (1.20-1.51)	<0.001	1.31 (1.15-1.48)	<0.001
**Primary psychiatric diagnosis** **Diagnosis categories**$ *(ICD-10 codes)*						
No primary psychiatric diagnosis recorded	8692 (32.6)	4258 (37.6)	1		1	
Schizophrenia, schizotypal and delusional disorders (F20-F29)	1818 (6.8)	522 (4.6)	0.59 (0.53-0.65)	<0.001	0.59 (0.53-0.67)	<0.001
Severe mood disorders (i.e. bipolar affective disorder, severe or moderate depressive disorders, puerperal psychosis and postnatal depression (F30-31, F32.1-32.3, F33.1-33.3, F34.0-34.1, F53.0-53.1)	535 (2.0)	246 (2.2)	0.94 (0.80-1.10)	0.424	0.96 (0.80-1.16)	0.698
Anxiety, somatoform and stress-related disorders (F40-48)	3061 (11.5)	1581 (14.0)	1.05 (0.98-1.13)	0.144	1.02 (0.94-1.10)	0.705
Other depressive disorders (F32.0, F32.8-32.9, F33.0, F33.4-33.9, F34.8-34.9, F38-39).	3711 (13.9)	1823 (16.1)	1.00 (0.94-1.07)	0.935	1.02 (0.94-1.10)	0.611
Drug and alcohol-related disorders (F10-19, excluding F17)	6240 (23.4)	1624 (14.3)	0.53 (0.50-0.57)	<0.001	0.59 (0.55-0.64)	<0.001
Personality disorders (F60-63)	658 (2.5)	235 (2.1)	0.73 (0.63-0.85)	<0.001	0.72 (0.60-0.85)	<0.001
Other psychiatric disorders (including eating disorders, other perinatal psychiatric disorders, and 'unspecified mental illness') (F50-3, F53.8-53.9, F99)	578 (2.2)	434 (3.8)	1.53 (1.35-1.75)	<0.001	1.38 (1.19-1.60)	<0.001
Intellectual disabilities (F70-F79)	153 (0.6)	12 (0.1)	0.16 (0.09-0.29)	<0.001	0.16 (0.09-0.29)	<0.001
Disorders of psychological development and behavioural and emotional disorders	1248 (4.7)	587 (5.2)	0.96 (0.86-1.07)	0.447	0.84 (0.75-0.94)	0.002

with onset usually occurring in childhood or adolescence (F80-89, F90-98)						
**Severe mental illness diagnosis** *(if yes to primary psychiatric diagnosis)*						
No severe mental illness diagnosis	14249 (78.5)	5655 (79.4)	1		1	
Severe mental illness diagnosis (F2* (schizophrenia-spectrum disorder), F30*/F31* (bipolar affective disorder) and F3* (affective disorder)	3909 (21.5)	1471 (20.6)	0.95 (0.89-1.01)	0.122	0.96 (0.89-1.04)	0.358

AOR: Adjusted Odds Ratio. IMD: Index of Multiple Deprivation. OR: Odds ratio. UC: Universal Credit. ^ Calculated at the UC window start date (January 2013). ¥ IMD scores published in 2015, patient postcode used closest before or after the UC window start date (January 2013). $ Earliest available within study window (January 2007 to December 2019), based on ICD-10 ‘F codes’ only (mental and behavioural disorders) but excluding non-specific diagnoses, for example, Z*, F99*, FXX. # Adjusted for age (continuous), sex, ethnicity, deprivation and primary psychiatric diagnosis (yes/no).

Supplementary Table 7: Overview of patients by UC conditionality regime – preparing for work (N=38701 of whom N=3812 were allocated to the UC – preparing for work regime). Odds ratio (OR), Adjusted odds ratios (AOR) and their corresponding 95% intervals (CI) represent increase in odds of UC receipt.

**Characteristics**	**Never allocated UC – preparing for work 2013-2019 N (%)**	**Allocated UC - preparing for work 2013-2019 N (%)**	**OR (95% CI)**	**p-value**	**AOR# (95% CI)**	**p-value**
**Sex**						
Female	15775 (45.2)	2224 (58.3)	1.70 (1.59-1.82)	<0.001	1.67 (1.55-1.80)	<0.001
Male	19114 (54.8)	1588 (41.7)	1		1	
**Age (years)^**						
18-24	8227 (23.6)	948 (24.9)	1		1	
25-34	11306 (32.4)	1252 (32.8)	0.96 (0.88-1.05)	0.381	0.93 (0.84-1.03)	0.152
35-44	8215 (23.6)	854 (22.4)	0.90 (0.82-0.99)	0.038	0.93 (0.84-1.04)	0.213
45-54	5965 (17.1)	623 (16.3)	0.91 (0.81-1.01)	0.070	0.91 (0.80-1.02)	0.099
55-66	1176 (3.4)	135 (3.5)	1.00 (0.82-1.21)	0.969	1.00 (0.81-1.23)	0.973
**Ethnicity**						
White	14931 (50.4)	1659 (51.0)	1		1	
Black/African/Caribbean/ Black British	6322 (21.3)	667 (20.5)	0.95 (0.86-1.04)	0.283	0.89 (0.81-0.98)	0.018
Asian/Asian British	718 (2.4)	85 (2.6)	1.07 (0.85-1.34)	0.590	1.05 (0.83-1.33)	0.671
Mixed/Multiple racial and ethnic groups	926 (3.1)	117 (3.6)	1.14 (0.93-1.39)	0.205	1.05 (0.86-1.29)	0.636
Other racial and ethnic minority groups	52 (0.2)	8 (0.3)	1.38 (0.66-2.92)	0.393	1.51 (0.71-3.20)	0.289
Not stated	6684 (22.6)	716 (22.0)	0.96 (0.88-1.06)	0.437	0.99 (0.90-1.10)	0.878
**Deprivation (IMD quintile)¥**						
First (most deprived)	11915 (36.2)	1418 (38.9)	1		1	

Second	11854 (36.1)	1354 (37.1)	0.96 (0.89-1.04)	0.307	0.94 (0.86-1.03)	0.170
Third	5487 (16.7)	584 (16.0)	0.89 (0.81-0.99)	0.031	0.84 (0.76-0.94)	0.003
Fourth	2305 (7.0)	195 (5.4)	0.71 (0.61-0.83)	<0.001	0.70 (0.59-0.83)	<0.001
Fifth (least deprived)	1317 (4.0)	96 (2.6)	0.61 (0.49-0.76)	<0.001	0.59 (0.47-0.74)	<0.001
**Primary psychiatric diagnosis** **Diagnosis categories**$ *(ICD-10 codes)*						
No primary psychiatric diagnosis recorded	11729 (34.2)	1221 (32.5)	1		1	
Schizophrenia, schizotypal and delusional disorders (F20-F29)	2246 (6.6)	94 (2.5)	0.40 (0.32-0.50)	<0.001	0.45 (0.35-0.57)	<0.001
Severe mood disorders (i.e. bipolar affective disorder, severe or moderate depressive disorders, puerperal psychosis and postnatal depression (F30-31, F32.1-32.3, F33.1-33.3, F34.0-34.1, F53.0-53.1)	717 (2.1)	64 (1.7)	0.86 (0.66-1.11)	0.251	0.88 (0.66-1.19)	0.415
Anxiety, somatoform and stress-related disorders (F40-48)	4076 (11.9)	566 (15.1)	1.33 (1.20-1.48)	<0.001	1.30 (1.15-1.47)	<0.001
Other depressive disorders (F32.0, F32.8-32.9, F33.0, F33.4-33.9, F34.8-34.9, F38-39).	4812 (14.1)	722 (19.2)	1.44 (1.31-1.59)	<0.001	1.36 (1.22-1.52)	<0.001
Drug and alcohol-related disorders (F10-19, excluding F17)	7121 (20.8)	743 (19.8)	1.00 (0.91-1.10)	0.963	1.13 (1.00-1.26)	0.041
Personality disorders (F60-63)	810 (2.4)	83 (2.2)	0.98 (0.78-1.24)	0.894	0.87 (0.67-1.14)	0.317
Other psychiatric disorders (including eating disorders, other perinatal psychiatric disorders, and ‘unspecified mental illness’) (F50-3, F53.8-53.9, F99)	905 (2.6)	107 (2.9)	1.14 (0.92-1.40)	0.232	0.88 (0.68-1.12)	0.302
Intellectual disabilities (F70-F79)	149 (0.4)	16 (0.4)	1.03 (0.61-1.73)	0.907	0.97 (0.56-1.70)	0.928
Disorders of psychological development and behavioural and emotional disorders with onset usually occurring in childhood or adolescence (F80-89, F90-98)	1691 (4.9)	144 (3.8)	0.82 (0.68-0.98)	0.029	0.92 (0.76-1.11)	0.393

**Severe mental illness diagnosis** *(if yes to primary psychiatric diagnosis)*						
No severe mental illness diagnosis	17786 (78.3)	2118 (82.5)	1		1	
Severe mental illness diagnosis (F2* (schizophrenia-spectrum disorder), F30*/F31* (bipolar affective disorder) and F3* (affective disorder)	4932 (21.7)	448 (17.5)	0.76 (0.69-0.85)	<0.001	0.77 (0.68-0.87)	<0.001

AOR: Adjusted Odds Ratio. IMD: Index of Multiple Deprivation. OR: Odds ratio. UC: Universal Credit. ^ Calculated at the UC window start date (January 2013). ¥ IMD scores published in 2015, patient postcode used closest before or after the UC window start date (January 2013). $ Earliest available within study window (January 2007 to December 2019), based on ICD-10 'F codes' only (mental and behavioural disorders) but excluding non-specific diagnoses, for example, Z*, F99*, FXX. # Adjusted for age (continuous), sex, ethnicity, deprivation and primary psychiatric diagnosis (yes/no).

Supplementary Table 8: Overview of patients by UC conditionality regime - planning for work (N=38701 of whom N=1823 were allocated to the UC – planning for work conditionality regime). Odds ratio (OR), Adjusted odds ratios (AOR) and their corresponding 95% intervals (CI) represent increase in odds of UC receipt.

**Characteristics**	**Never allocated to UC – planning for work 2013-2019 N (%)**	**Allocated to UC – planning for work 2013-2019 N (%)**	**OR (95% CI)**	**p-value**	**AOR# (95% CI)**	**p-value**
**Sex**						
Female	16434 (44.6)	1565 (85.9)	7.55 (6.60-8.62)	<0.001	6.81 (5.86-7.91)	<0.001
Male	20444 (55.4)	258 (14.2)	1		1	
**Age (years)**^						
18-24	8378 (22.7)	797 (43.7)	1		1	
25-34	11715 (31.8)	843 (46.2)	0.76 (0.68-0.84)	<0.001	0.77 (0.69-0.87)	<0.001
35-44	8910 (24.2)	159 (8.7)	0.19 (0.16-0.22)	<0.001	0.18 (0.15-0.22)	<0.001
45-54	6567 (17.8)	21 (1.2)	0.03 (0.02-0.05)	<0.001	0.03 (0.02-0.06)	<0.001
55-66	1308 (3.6)	<5 (<1.0)	0.02 (0.01-0.08)	<0.001	0.03 (0.01-0.10)	<0.001
**Ethnicity**						
White	15958 (50.9)	632 (41.9)	1		1	
Black/African/Caribbean/ Black British	6570 (20.9)	419 (27.8)	1.61 (1.42-1.83)	<0.001	1.37 (1.20-1.57)	<0.001
Asian/Asian British	757 (2.4)	46 (3.1)	1.53 (1.13-2.09)	0.007	1.41 (1.02-1.94)	0.038
Mixed/Multiple racial and ethnic groups	987 (3.2)	56 (3.7)	1.43 (1.08-1.90)	0.012	0.90 (0.67-1.20)	0.464
Other racial and ethnic minority groups	57 (0.2)	<5 (<1.0)	1.33 (0.42-4.25)	0.632	1.48 (0.43-5.07)	0.534
Not stated	7048 (22.5)	352 (23.3)	1.26 (1.10-1.44)	0.001	1.13 (0.98-1.31)	0.087
**Deprivation (IMD quintile)**¥						

First (most deprived)	12641 (36.4)	692 (39.1)	1		1	
Second	12535 (36.1)	673 (38.0)	0.98 (0.88-1.09)	0.727	0.98 (0.87-1.11)	0.760
Third	5807 (16.7)	264 (14.9)	0.83 (0.72-0.96)	0.012	0.79 (0.67-0.94)	0.006
Fourth	2402 (6.9)	98 (5.5)	0.75 (0.60-0.92)	0.008	0.71 (0.55-0.90)	0.005
Fifth (least deprived)	1368 (3.9)	45 (2.5)	0.60 (0.44-0.82)	0.001	0.55 (0.39-0.78)	0.001
**Primary psychiatric diagnosis** **Diagnosis categories**$ *(ICD-10 codes)*						
No primary psychiatric diagnosis recorded	12276 (33.9)	674 (37.3)	1		1	
Schizophrenia, schizotypal and delusional disorders (F20-F29)	2292 (6.3)	48 (2.7)	0.38 (0.28-0.51)	<0.001	0.45 (0.32-0.64)	<0.001
Severe mood disorders (i.e. bipolar affective disorder, severe or moderate depressive disorders, puerperal psychosis and postnatal depression (F30-31, F32.1-32.3, F33.1-33.3, F34.0-34.1, F53.0-53.1)	756 (2.1)	25 (1.4)	0.60 (0.40-0.90)	0.014	0.71 (0.45-1.12)	0.137
Anxiety, somatoform and stress-related disorders (F40-48)	4354 (12.0)	288 (15.9)	1.20 (1.05-1.39)	0.010	1.01 (0.85-1.19)	0.924
Other depressive disorders (F32.0, F32.8-32.9, F33.0, F33.4-33.9, F34.8-34.9, F38-39).	5123 (14.2)	411 (22.7)	1.46 (1.29-1.66)	<0.001	1.30 (1.12-1.52)	0.001
Drug and alcohol-related disorders (F10-19, excluding F17)	7706 (21.3)	158 (8.7)	0.37 (0.31-0.45)	<0.001	0.77 (0.62-0.94)	0.012
Personality disorders (F60-63)	854 (2.4)	39 (2.2)	0.83 (0.60-1.16)	0.274	0.64 (0.44-0.94)	0.023
Other psychiatric disorders (including eating disorders, other perinatal psychiatric disorders, and ‘unspecified mental illness’) (F50-3, F53.8-53.9, F99)	934 (2.6)	78 (4.3)	1.52 (1.19-1.94)	0.001	0.87 (0.65-1.16)	0.344
Intellectual disabilities (F70-F79)	161 (0.4)	<5 (<1.0)	0.45 (0.17-1.22)	0.118	0.48 (0.17-1.33)	0.158
Disorders of psychological development and behavioural and emotional disorders with	1751 (4.8)	84 (4.6)	0.87 (0.69-1.10)	0.255	0.82 (0.64-1.06)	0.126

onset usually occurring in childhood or adolescence (F80-89, F90-98)						
**Severe mental illness diagnosis** *(if yes to primary psychiatric diagnosis)*						
No severe mental illness diagnosis	18992 (78.7)	912 (79.3)	1		1	
Severe mental illness diagnosis (F2* (schizophrenia-spectrum disorder), F30*/F31* (bipolar affective disorder) and F3* (affective disorder)	5142 (21.3)	238 (20.7)	0.96 (0.83-1.12)	0.621	0.96 (0.81-1.14)	0.638

AOR: Adjusted Odds Ratio. IMD: Index of Multiple Deprivation. OR: Odds ratio. UC: Universal Credit. ^ Calculated at the UC window start date (January 2013). ¥ IMD scores published in 2015, patient postcode used closest before or after the UC window start date (January 2013). $ Earliest available within study window (January 2007 to December 2019), based on ICD-10 ‘F codes’ only (mental and behavioural disorders) but excluding non-specific diagnoses, for example, Z*, F99*, FXX. # Adjusted for age (continuous), sex, ethnicity, deprivation and primary psychiatric diagnosis (yes/no).

Supplementary Table 9: Overview of sociodemographic and diagnostic patient characteristics and UC receipt (irrespective of conditionality regime) between 2013-2019, restricted to patients who lived in the South London and Maudsley NHS Foundation Trust catchment area (N=95661 of whom N=27648 had received UC). Odds ratio (OR), Adjusted odds ratios (AOR) and their corresponding 95% intervals (CI) represent increase in odds of UC receipt.

**Characteristics**	**Not received UC N (%)**	**Received UC N (%)**	**OR (95% CI)**	**p-value**	**AOR# (95% CI)**	**p-value**
**Sex**						
Female	35049 (51.4)	13865 (50.5)	0.96 (0.94-0.99)	0.010	0.92 (0.89-0.95)	<0.001
Male	33144 (48.6)	13603 (49.5)	1		1	
**Age (years)^**						
18-24	9069 (13.3)	6062 (22.1)	1		1	
25-34	18606 (27.3)	8739 (31.8)	0.70 (0.67-0.73)	<0.001	0.71 (0.68-0.75)	<0.001
35-44	17742 (26.0)	6544 (23.8)	0.55 (0.53-0.58)	<0.001	0.55 (0.52-0.57)	<0.001
45-54	17347 (25.4)	5075 (18.5)	0.44 (0.42-0.46)	<0.001	0.41 (0.39-0.43)	<0.001
55-66	5429 (8.0)	1048 (3.8)	0.29 (0.27-0.31)	<0.001	0.28 (0.26-0.30)	<0.001
**Ethnicity**						
White	29312 (54.3)	10891 (48.1)	1		1	
Black/African/Caribbean/ Black British	11642 (21.6)	6104 (27.0)	1.41 (1.36-1.47)	<0.001	1.33 (1.28-1.38)	<0.001
Asian/Asian British	1615 (3.0)	618 (2.7)	1.03 (0.94-1.13)	0.545	0.98 (0.89-1.08)	0.680
Mixed/Multiple racial and ethnic groups	1542 (2.9)	854 (3.8)	1.49 (1.37-1.63)	<0.001	1.25 (1.14-1.37)	<0.001
Other racial and ethnic minority groups	93 (0.2)	45 (0.2)	1.30 (0.91-1.86)	0.147	1.24 (0.86-1.78)	0.255
Not stated	9793 (18.1)	4126 (18.2)	1.13 (1.09-1.18)	<0.001	1.04 (1.00-1.09)	0.072
**Deprivation (IMD quintile)¥**						
First (most deprived)	23813 (35.3)	11108 (41.2)	1		1	
Second	25940 (38.4)	10295 (38.2)	0.85 (0.82-0.88)	<0.001	0.85 (0.82-0.88)	<0.001
Third	12350 (18.3)	4056 (15.1)	0.70 (0.68-0.73)	<0.001	0.70 (0.67-0.74)	<0.001

Fourth	3689 (5.5)	1012 (3.8)	0.59 (0.55-0.63)	<0.001	0.60 (0.55-0.65)	<0.001
Fifth (least deprived)	1682 (2.5)	475 (1.8)	0.61 (0.55-0.67)	<0.001	0.61 (0.55-0.69)	<0.001
**Primary psychiatric diagnosis** **Diagnosis categories**$ *(ICD-10 codes)*						
No primary psychiatric diagnosis recorded	19767 (29.8)	8393 (31.1)	1		1	
Schizophrenia, schizotypal and delusional disorders (F20-F29)	6512 (9.8)	1984 (7.4)	0.72 (0.68-0.76)	<0.001	0.64 (0.59-0.68)	<0.001
Severe mood disorders (i.e. bipolar affective disorder, severe or moderate depressive disorders, puerperal psychosis and postnatal depression (F30-31, F32.1-32.3, F33.1-33.3, F34.0-34.1, F53.0-53.1)	2385 (3.6)	646 (2.4)	0.64 (0.58-0.70)	<0.001	0.66 (0.59-0.73)	<0.001
Anxiety, somatoform and stress-related disorders (F40-48)	9184 (13.8)	3640 (13.5)	0.93 (0.89-0.98)	0.003	0.93 (0.88-0.98)	0.010
Other depressive disorders (F32.0, F32.8-32.9, F33.0, F33.4-33.9, F34.8-34.9, F38-39).	12492 (18.8)	4954 (18.4)	0.93 (0.90-0.97)	0.001	0.96 (0.92-1.01)	0.137
Drug and alcohol-related disorders (F10-19, excluding F17)	8675 (13.1)	5029 (18.7)	1.37 (1.31-1.43)	<0.001	1.63 (1.54-1.71)	<0.001
Personality disorders (F60-63)	1494 (2.3)	690 (2.6)	1.09 (0.99-1.19)	0.079	1.08 (0.97-1.21)	0.138
Other psychiatric disorders (including eating disorders, other perinatal psychiatric disorders, and ‘unspecified mental illness’) (F50-3, F53.8-53.9, F99)	2869 (4.3)	710 (2.6)	0.58 (0.53-0.64)	<0.001	0.51 (0.46-0.56)	<0.001
Intellectual disabilities (F70-F79)	1429 (2.2)	148 (0.6)	0.24 (0.21-0.29)	<0.001	0.23 (0.19-0.28)	<0.001
Disorders of psychological development and behavioural and emotional disorders with onset usually occurring in childhood or adolescence (F80-89, F90-98)	1625 (2.5)	755 (2.8)	1.09 (1.00-1.20)	0.050	0.82 (0.74-0.90)	<0.001
**Severe mental illness diagnosis** *(if yes to primary psychiatric diagnosis)*						

No severe mental illness diagnosis	33194 (70.8)	14092 (75.3)	1		1	
Severe mental illness diagnosis (F2* (schizophrenia-spectrum disorder), F30*/F31* (bipolar affective disorder) and F3* (affective disorder)	13715 (29.2)	4612 (24.7)	0.79 (0.76-0.82)	<0.001	0.74 (0.71-0.77)	<0.001

AOR: Adjusted Odds Ratio. IMD: Index of Multiple Deprivation. OR: Odds ratio. UC: Universal Credit. ^ Calculated at the UC window start date (January 2013). ¥ IMD scores published in 2015, patient postcode used closest before or after the UC window start date (January 2013). $ Earliest available within study window (January 2007 to December 2019), based on ICD-10 ‘F codes’ only (mental and behavioural disorders) but excluding non-specific diagnoses, for example, Z*, F99*, FXX. # Adjusted for age (continuous), sex, ethnicity, deprivation and primary psychiatric diagnosis (yes/no).

Supplementary Table 10: Overview of sociodemographic and diagnostic patient characteristics (weighted percentages) and UC receipt and weighted logistic regression analyses results (irrespective of conditionality regime) between 2013-2019 (N=140155 of whom N=37752 had received UC). Odds ratio (OR), Adjusted odds ratios (AOR) and their corresponding 95% intervals (CI) represent increase in odds of UC receipt.

**Characteristics**	**Not received UC N (%)**	**Received UC N (%)**	**OR (95% CI)**	**p-value**	**AOR# (95% CI)**	**p-value**
**Sex**						
Female	50316 (49.7)	17583 (47.3)	0.91 (0.89-0.93)	<0.001	0.87 (0.85-0.90)	<0.001
Male	52087 (50.3)	20169 (52.7)	1		1	
**Age (years)**^						
18-24	15102 (15.0)	8974 (24.1)	1		1	
25-34	27989 (27.8)	12242 (32.8)	0.73 (0.71-0.76)	<0.001	0.73 (0.70-0.76)	<0.001
35-44	26493 (25.8)	8818 (23.1)	0.56 (0.54-0.58)	<0.001	0.54 (0.52-0.56)	<0.001
45-54	25207 (24.4)	6448 (16.9)	0.43 (0.41-0.45)	<0.001	0.39 (0.38-0.41)	<0.001
55-66	7612 (7.1)	1270 (3.2)	0.28 (0.26-0.30)	<0.001	0.27 (0.25-0.29)	<0.001
**Ethnicity**						
White	48664 (55.2)	16590 (49.4)	1		1	
Black/African/Caribbean/ Black British	13525 (16.2)	6989 (22.1)	1.52 (1.47-1.57)	<0.001	1.39 (1.34-1.44)	<0.001
Asian/Asian British	2320 (2.8)	803 (2.5)	1.02 (0.94-1.10)	0.699	0.95 (0.88-1.04)	0.273
Mixed/Multiple racial and ethnic groups	1997 (2.3)	1043 (3.2)	1.53 (1.42-1.65)	<0.001	1.28 (1.18-1.38)	<0.001
Other racial and ethnic minority groups	132 (0.2)	60 (0.2)	1.33 (0.98-1.81)	0.065	1.13 (0.81-1.58)	0.458
Not stated	19967 (23.3)	7400 (22.7)	1.09 (1.05-1.12)	<0.001	1.00 (0.97-1.04)	0.876
**Deprivation (IMD quintile)¥**						
First (most deprived)	29060 (29.8)	13009 (36.6)	1		1	
Second	33824 (34.6)	12857 (36.1)	0.85 (0.82-0.87)	<0.001	0.85 (0.83-0.88)	<0.001
Third	19015 (19.4)	5920 (16.6)	0.70 (0.67-0.72)	<0.001	0.69 (0.67-0.72)	<0.001
Fourth	9718 (9.9)	2457 (6.8)	0.56 (0.54-0.59)	<0.001	0.56 (0.53-0.59)	<0.001

Fifth (least deprived)	6323 (6.4)	1382 (3.8)	0.49 (0.46-0.52)	<0.001	0.49 (0.46-0.52)	<0.001
**Primary psychiatric diagnosis**<br>**Diagnosis categories**$ *(ICD-10 codes)*						
No primary psychiatric diagnosis recorded	33042 (33.3)	12671 (34.3)	1		1	
Schizophrenia, schizotypal and delusional disorders (F20-F29)	7987 (8.1)	2272 (6.2)	0.75 (0.71-0.79)	<0.001	0.64 (0.60-0.67)	<0.001
Severe mood disorders (i.e. bipolar affective disorder, severe or moderate depressive disorders, puerperal psychosis and postnatal depression (F30-31, F32.1-32.3, F33.1-33.3, F34.0-34.1, F53.0-53.1)	3057 3.1)	751 (2.0)	0.64 (0.59-0.70)	<0.001	0.68 (0.61-0.74)	<0.001
Anxiety, somatoform and stress-related disorders (F40-48)	13717 (13.8)	4466 (12.1)	0.85 (0.82-0.89)	<0.001	0.87 (0.83-0.91)	<0.001
Other depressive disorders (F32.0, F32.8-32.9, F33.0, F33.4-33.9, F34.8-34.9, F38-39).	14456 (14.6)	5357 (14.5)	0.97 (0.93-1.01)	0.113	1.00 (0.96-1.04)	0.952
Drug and alcohol-related disorders (F10-19, excluding F17)	14413 (14.2)	7729 (20.5)	1.40 (1.35-1.45)	<0.001	1.63 (1.56-1.70)	<0.001
Personality disorders (F60-63)	2063 (2.1)	882 (2.4)	1.11 (1.03-1.21)	0.010	1.07 (0.97-1.17)	0.168
Other psychiatric disorders (including eating disorders, other perinatal psychiatric disorders, and 'unspecified mental illness') (F50-3, F53.8-53.9, F99)	4562 (4.6)	992 (2.7)	0.57 (0.53-0.61)	<0.001	0.53 (0.48-0.57)	<0.001
Intellectual disabilities (F70-F79)	1551 (1.6)	161 (0.4)	0.27 (0.23-0.32)	<0.001	0.25 (0.21-0.29)	<0.001
Disorders of psychological development and behavioural and emotional disorders with onset usually occurring in childhood or adolescence (F80-89, F90-98)	4821 (4.8)	1817 (4.9)	0.98 (0.92-1.04)	0.486	0.82 (0.77-0.88)	<0.001
**Severe mental illness diagnosis** *(if yes to primary psychiatric diagnosis)*						
No severe mental illness diagnosis	50301 (74.9)	19436 (78.7)	1		1	

Severe mental illness diagnosis (F2* (schizophrenia-spectrum disorder), F30*/F31* (bipolar affective disorder) and F3* (affective disorder)	16747 (25.1)	5207 (21.4)	0.81 (0.78-0.84)	<0.001	0.74 (0.71-0.77)	<0.001

AOR: Adjusted Odds Ratio. IMD: Index of Multiple Deprivation. OR: Odds ratio. UC: Universal Credit. ^ Calculated at the UC window start date (January 2013). ¥ IMD scores published in 2015, patient postcode used closest before or after the UC window start date (January 2013). $ Earliest available within study window (January 2007 to December 2019), based on ICD-10 ‘F codes’ only (mental and behavioural disorders) but excluding non-specific diagnoses, for example, Z*, F99*, FXX. # Adjusted for age (continuous), sex, ethnicity, deprivation and primary psychiatric diagnosis (yes/no).

Supplementary Table 11: Overview of Severe Mental Illness diagnosis and UC receipt (irrespective of conditionality regime) between 2013-2019, restricted to patients who had not received legacy benefits (N=22945 of whom N=3269 had received UC). Odds ratio (OR), Adjusted odds ratios (AOR) and their corresponding 95% intervals (CI) represent increase in odds of UC receipt.

**Characteristics**	**Not received UC N (%)**	**Received UC N (%)**	**OR (95% CI)**	**p-value**	**AOR# (95% CI)**	**p-value**
**Severe mental illness diagnosis** *(if yes to primary psychiatric diagnosis)*						
No severe mental illness diagnosis	15604 (79.3)	2439 (74.6)	1		1	
Severe mental illness diagnosis (F2* (schizophrenia-spectrum disorder), F30*/F31* (bipolar affective disorder) and F3* (affective disorder)	4072 (20.7)	830 (25.4)	1.30 (1.20-1.42)	<0.001	1.12 (1.00-1.24)	0.045

CI: Confidence Interval. UC: Universal Credit. # Adjusted for age (continuous), sex, ethnicity, deprivation and primary psychiatric diagnosis (yes/no).
